# Barriers to and Facilitators of Road Traffic Injuries Prevention in Iran; A Qualitative Study

**DOI:** 10.29252/beat-070408

**Published:** 2019-10

**Authors:** Saber Azami-Aghdash, Hassan Abolghasem Gorji, Naser Derakhshani, Homayoun Sadeghi-Bazargani

**Affiliations:** 1 *Tabriz Health Services Management Research Center, Health* *Management and Safety Promotion Research Institute, Tabriz* *University of Medical Sciences, Tabriz, Iran.*; 2 *School of health management and information sciences, Iran University of Medical Sciences, Tehran, Iran.*; 3 *Health management and Economics Research Center, Iran University of Medical Sciences, Tehran, Iran*; 4 *Road Traffic Injury Research Center, Tabriz University of Medical Sciences, Tabriz, Iran*

**Keywords:** Road traffic injuries, Prevention, Barriers, Facilitators.

## Abstract

**Objective:**

To identify the barriers to and facilitators of the prevention of road traffic accidents (RTIs) in Iran.

**Methods:**

In this qualitative study 42 key stakeholders and experts in the field of traffic injuries in Iran were selected based on purpose and theoretical sampling to reach informational saturation. Their views concerning barriers to and facilitators of the prevention of traffic injuries in Iran were studied using semi-structured interviews. Data were analyzed using Content Analysis method.

**Results:**

Five themes were identified: structural barriers, organizational barriers and planning, socio-cultural barriers, scientific barriers, and inter-sector barriers and 22 sub-themes were extracted. The lack of lead agency, which was among structural barriers, was selected as the main barrier. The five general facilitators included: sensitization of society and authorities, improving the resources and infrastructure (software and hardware), increasing the attention to safety of vehicles and roads, increasing the information and awareness, and use of expert manpower. The sensitization of society and authorities was selected as the most important facilitator.

**Conclusion:**

According to the key experts, the barriers of policy changes to prevent the RTIs are more numerous than its facilitators. Therefore, planning and paying more attention to removing these barriers and promoting the facilitators seems necessary to reduce RTIs. Having a lead agency in this regard should be the highest priority.

## Introduction

Road traffic injuries (RTIs) are considered one of the main causes of mortality and disability in the world [1, 2]. Most of the hospitalizations in the emergency departments of the hospitals are due to RTIs that impose enormous costs on the government and people [3, 4]. Annually 1.2 million people throughout the world lose their lives due to RTIs and 50 million became injured [5]. It is estimated that 92 percent of the RTIs’ mortality occur in in Low and middle Income Countries (LMICs) [6]. According to the World Health Organization (WHO), the RTIs in Iran led to 24896 deaths in a year (according to national calendar covering April 2013- April 2014). Of which three quarters were men.  So the related mortality rate per 100 thousand people in Iran was 32.1 [5]. From a historical standpoint, RTIs are considered as accidents occurred to others and as inevitable and non-preventable events. The word “accident” is especially reminiscent of being non-preventable, while results of research and high agreement between the experts show that the RTIs are preventable [7-10]. In the High Income Countries (HICs), a set of measures to prevent RTIs have been taken. These measures include legislation to control speed and alcohol consumption, use of safety belts and helmets, designing safer roads and vehicles [11, 12].

 RTIs are the leading cause of injuries and the second cause of mortality in Iran [13-16], and it is 4 times more prevalent than the HICs [17]. Death rate due to RTIs in Iran also is highest as compared to other LMICs [18-20]. The RTIs in Iran cause loss of 2271 years of life annually and damage amounting to 6 billion dollars [21, 22]. From 2005 to 2008, Iran led the word in highest number of deaths caused by RTIs[23, 24]. At that time, the study of Khorasani-Zavareh *et al*. (2009) showed that there were many barriers in Iran to prevent RTIs [25]. Since then, in Iran, like many LMICs, various interventions and programs were designed and implemented to prevent the RTIs [26-30]. So the WHO’s report in 2015 shows that mortality caused by RTIs in Iran has declined from approximately 40 per 100 thousand people in 2005 to approximately 32.1 per 100 thousand people in 2014 [5].

Despite progress in the field of prevention of RTIs in recent years, traffic incidents in Iran are still the main cause of injuries and the second cause of mortality [19, 31-33]. Therefore, it seems that there are still some barriers (adapting effective policies or interventions and their implementation) in the prevention of RTIs that require further study in this regard. On the other hand, for better management and for adjusting the past failures, investigating the facilitator of the prevention of RTIs seems indispensable. To identify the barriers and facilitators, examining experts’ experiences and views is one of the most suitable solutions [34-36]. Thus, the aim of the present study was to identify the barriers and facilitators of the prevention of RTIs in Iran.

## Materials and Methods

This qualitative study conducted in 2016. The reason for using the qualitative method was the ability of these studies in drawing out the participants’ experiences, knowledge and unraveled information [37, 38]. Among the various approaches of qualitative studies, the conventional Content-Analysis approach was selected. This approach is an appropriate approach to collect valid and reliable results; that can be used to obtain and develop rich information, insights and a guide to performance [39]. Conventional Content analysis is a method that has become popular in health studies recently. The content analysis is a flexible method for analyzing qualitative data. It describes a group of analytic approaches that differ from impressionistic, intuitive, interpretive analyses to systematic, strict textual analyses. Advantage of the conventional approach to content analysis is getting direct information from the participants without imposing preconceived categories or theoretical perspectives [40].

Key informants were selected from the following institutions: Ministry of Roads and Transportation, Ministry of Industries, Ministry of Health and Medical Education, Traffic Police, Ministry of Islamic Culture and Guidance, Ministry of Education, Legal Medicine, Organization (forensic medicine), Insurance Organization, Ministry of Justice, Ministry of Interior, the Red Crescent Organization, Emergency Services, Firefighting, Police, Broadcasting Organization and the Judiciary branch. The study participants were faculty members who had extensive research on RTIs, heads of research centers related to RTIs, directors or senior officials in the mentioned organizations who had direct responsibility for RTIs and its prevention and a number of literate drivers. These individuals were selected since they had considerable experience and knowledge concerning the prevention of RTIs. The inclusion criteria included having published books, articles or other research works on RTIs for faculty members and heads of research centers, having at least two years of experience in jobs related to RTIs, Iranian nationality, the Persian language proficiency, diploma degree, and having the will and ability to participate in the study.

The study began with purposeful sampling method and then the theatrical sampling method was applied. In this method, some individuals are selected as participants who have the largest and richest information and are able to provide the researchers with their information appropriately [41-43]. It continued till data saturation, it means reaching a point where the researchers feel the new information does not come with the arrival of new people. The saturation was achieved with 42 participants. After the start of the study and during the interviews, theoretical sampling was used to identify people who could provide the researchers with rich and useful information. In addition, it was attempted to have various participants in terms of age, employment status, work experience, education and job status to create diverse data.

To collect the data, semi-structured interviews were conducted in Persian. The interviews were conducted in a place that was convenient for the participants. During the interviews, the guide questions, which were designed using the literature review and experts’ views, were used (appendex1). The duration of each interview varied between 45 to 90 minutes (except one case which due to high business, it ended in 20 minutes). The individuals’ statements were recorded with their consent by using a tape recorder and the research also used note taking during the interviews. The interviews immediately after the end of each interview was reviewed by the researchers several times and typed in Microsoft Word. For four participants due to access problems (being abroad at the time of the study) open-ended questionnaire was used rather than the interview. The questionnaires were sent via email.

For data analysis, content-analysis method was applied, which is a method to identify, analyze and report patterns (themes) within the text. This type of analysis is used when the theories on the subject are limited [44-46]. Data analysis and coding processes were as follows: familiarizing with the data text (reading the transcripts several time-data immersion), identifying and extracting the basic codes (identifying and extracting more relevant data with the primary codes), identifying themes (placing the initial extracted codes in related classes and themes), reviewing and completing the identified themes, naming and defining themes, re-coding and renaming some classes and themes, and ensuring reliability of the codes.

To increase the rigor and accuracy of the findings, the four measures proposed by Goba and Lincoln [13] were used as below. For the Credibility and Confirmability, the researchers were involved long time with the data. Also the peer review and expert review were applied. The respondent validation used after each session as the summarized statements of the participant was told to him/her to affirm and to prevent mis-understandings. For Dependability aspect the coding was done by two coders. And for the Transferability, the expert review, purposeful sampling, and heterogeneous sampling were used. 

To observe ethical issues in this study, informed consent was obtained from the participants and they had the right to withdraw and leave the study at any time they want. In addition, the objectives of the study were explained to the participants before interviews. Ethical approval was obtained from the Ethics Committee in Research located at the Iran University of Medical Sciences. All the participants provided their informed written consents.

## Results

**Fig. 1:**
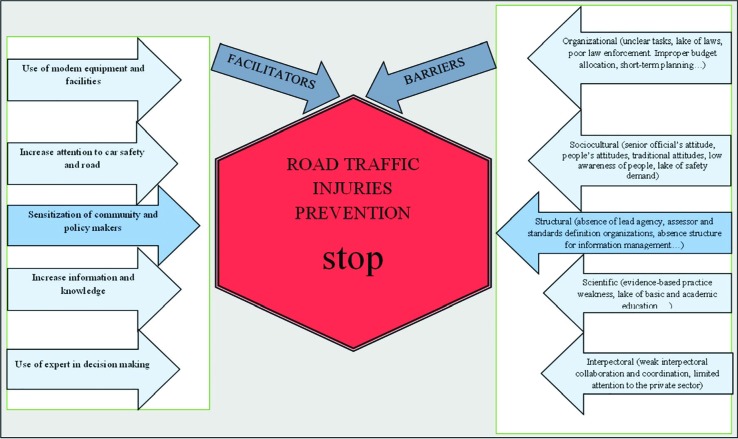
Barriers to and facilitators of the prevention of RTIs in Iran from the participants’ perspective

Among the 42 participants of the study, 9 were faculty members, which 4 of whom were the head of research centers, 20 were the senior officials of stakeholder organizations in the field of RTIs in the country, 10 were people who had responsibilities regarding the RTIs in the province, and 3 were literate and experienced drivers. Moreover, 23 participants had PhD education. The results of analyzing and coding the interviews include 5 main themes and 22 sub-themes regarding the barriers and 5 themes concerning the facilitators of the prevention of RTIs, which are shown in Figure 1.

In respect to the importance and the large number of barriers that participants highlighted, in this figure, the barriers are shown greater than the facilitators. The person in black who pushes the barriers is shown as a sign of “being negative” and the person in white who pushes facilitators is shown as a sign of “being positive”. The lack of traffic organization that is among the structural barriers, due to high repetition by the participants (almost all participants) and its importance was selected as the main barrier and is shown in red due to the consequences of failure to solve the problem (danger sign). In addition, the facilitator of “sensitiveness of society and authorities” is shown as the main theme of the facilitators and in green (a sign of good effect).

*Barriers*

5 main themes and 22 sub-themes on barriers to prevent RTIs were extracted. The full lists of the barriers with the participants’ direct quotes are summarized in Table 1. The lack of traffic lead agency that is among the structural barriers, due to high repetition by the participants (almost all participants) and its importance was selected as the main barrier. All participants pointed out that the lack of traffic lead agency with authority and executive power as well as adequate resources is the main problem of the prevention of RTIs in Iran. Most participants believed that an organization with the leadership of the country’s top officials should be responsible for the prevention of RTIs.

*“... In our country, in contrast to the successful countries in this regard, no one organization is in charge of RTIs and preventing them ... any organization does its work ... Nobody follows another one...." (P11).* *“... In our country, we have no organization with enough power and resources to lead the prevention of RTIs.... I think that the President or even the Supreme Leader should do something in the regard... “(P21).*

*Facilitators*

In this study, 5 general facilitators were extracted including sensitization of society and the authorities, improving the resources and infrastructure (software and hardware), increasing the attention to safety of vehicles and roads, increasing the information and awareness, and use of expert manpower. The sensitization of society and the authorities was selected as the main theme of the facilitators.

**Table 1:** Barriers to preventing RTIs in Iran from the participants’ perspective (N = 32)

**Main themes**	**Sub-themes**	**Direct quotations participants**
**Structural barriers**	Lack of responsible/ lead agency+	“... Nobody is in charge of the work (to prevent RTIs)...” P3 “... I want to say that I didn’t see the leading and responsible with sufficient resources and authority in our country...” P5
	Lack of evaluating organization	“... No monitoring organization claims against them and be accountable for it ....” P1
	Lack of informational structure	“... We still have not an informational structure that can collect, analyze and report timely and correctly...” P22
	Lack of an organization for determining standards	“... The Standard Organization of the country and even the Ministry of Health ,which are in charge of setting health standards, in setting the standards do not consider safety standards, especially vehicles and roads...” P12
**Organizational barriers and planning**	Unclearness of tasks	“... We have no road map, individuals’ role are unclear, what? ...” P1
	Lack of laws	“... I think we haven’t enough rules, in some areas there are still no certain rules... ” P27
	Misallocation of funds	“...I don’t say the main, but one of the problems of this sector is the misallocation of funds to the sector (to prevent RTIs)...” P27
	Weak law enforcement	“... In addition to the lack of legislation that I mentioned, I think that the existing rules are applied weakly and have weaknesses...” P27
	Short-term planning	“.. We have sectional planning, we have never had long-term thinking and strategic thinking...” P9
	Wrong priorities	“... How much of the funding of research is in the field of road traffic injuries, too little, the same is true in education....” P7
	Little attention to rail and sea transport systems	“... We have neglected the rail transport system ... When I sit in the train safely and cross the road at high speed from the car, surly, I use train...” P17 “... Unfortunately, in our country, public transportation including rail and naval transportations is not considered...” P15
**Socio-cultural barriers**	Senior officials’ attitude	“... One of the problems that we have, that the very senior officials in the country do not feel the need to participate or they are not aware of the importance of participation or due to too busy.... they did not have sufficient opportunity to enter this issues (prevention of RTIs)... “P27 “... When the Minister says that our problem is culture and police must solve this problem, people think that it is a projection ... when the Minister gives such an interview, we shouldn’t expect to solve the problem of RTIs...” P26
	People’s attitudes	“People’s attitudes to the law, legality and the person who enforces the law need to be approved...” P23
	Traditional view	“... One of our problems is ... our classical view to the accident so that accident is still seen as fate ...” P5
	People’s low awareness	“... Our people do not have knowledge in the field of safety and RTIs, ... perhaps it is our fault (our purpose is related authorities) that we did not give awareness ...” P31
	Low demand for safety	“... In this area, we have the lack of demand.... People’s demand must increase ...” P27
**Scientific barriers**	Weak evidence-based practice	“... We never could use the evidence properly.... Our managers do not pay attention to the evidence ...” P13
	Lack of basic education and academic	“... In our country, not only in the education system discussing traffic safety education has been neglected, but in the university and academic system, this issue is ignored ... We do not have enough education ... specialized fields should be created ... “ P23
	False benchmarking	“... We do not act properly in our benchmarking ... We see something on the news or in high-income countries and implement it in our country regardless of the our circumstances...”P27
	Weakness in the transfer of knowledge	“...The little knowledge that is sometimes produced, especially in academia, is not transmitted correctly to the users of information ...” P24
**Intersectional barriers**	Weak intersectional cooperation and coordination	“... The lack of coordination among the organizations…I want to say lack of cooperation... unfortunately, our organizations addition to the lack of coordination, have the lack of cooperation...” P29
	Little attention to the private sector	“... We could not take advantage of the capacity of the private sector appropriately...” P16

*Sensitization of Society and the Authorities*

Most participants acknowledged that due to the increase in the number of mortality and injuries resulting from RTIs, reports should be published at national and international level concerning RTIs in Iran. By publishing and broadcasting reports of RTIs, especially during the New Year period on the severity of the issue, both authorities and people became sensitized to the issue of RTIs and may consider it. Therefore, RTIs were on the agenda of many organizations and relevant authorities.

*“... In a particular period that RTIs increased and reached its peak, officials were sensitive to this issue and were concerned about the issue ...” (P13). “...In recent years, reports that the WHO released about RTIs and reflected the sad state of our country .... The reports that were published in our country... Dr. Naqvi’s report showed that RTIs cause the greatest burden of disability... they sensitized society and the authorities ...” (P16). “...The pictures and interviews that the TV shows ... bolded this issue ...” (P23).*

**Improving the resources and infrastructure (software and hardware) **Some of the participants mentioned the increase of the facilities and the necessity of updating them. Most participants considered this increase of facilities and equipment related to the traffic police. Among the most noted cases, violation-record camera, use of more equipped police cars, increasing the police budget and manpower, and other cases.

*“... In recent years, traffic police have good facilities such as suitable cars, violation-record camera and so on ...”* *(P31).* *“... Fortunately, with the assistance of the state in these years both budget and staffing have been improved in the traffic police...”* *(P21*).

*Increasing the Attention to Safety of Vehicles and Roads*

Most participants have noted the increase of the safety of roads and vehicles in recent years. Although most of the participants mentioned this point, majority of them did not consider the improvement sufficient enough and expressed that the status of vehicle and road is not comparable to the HICs and even many LMICs.

*“...* *Naturally, besides these, the relative increase of vehicle safety, including applying ABSs,* *air bags* and so on *was effective in reducing RTIs.... but they are not enough and we have a long way to reach the level of advanced countries and even many* *of the developing world ...” (P11).* *“...* *In addition, road safety is also improved over the years ...* *but a lot of defects still remain...” (P8*).

*Increasing the Information and Awareness*

Some participants mentioned the role of the increased information and awareness on the prevention of RTIs. The increase of research centers, the number of professors and researchers in the field, reports released by international institutions such as the WHO and the World Bank, holding conferences, broadcasting activities and other causes were among the cases that participants noted as the factors of the increasing the awareness and information.

*“... Fortunately, in recent years, many research centers have spread... new professors and researchers are entered to the field... many risk factors have been identified ... many detailed reports were published ...”* *(P18*).

*Use of Expert Manpower*

The use of educated and expert manpower in decision making and executive offices was among the other cases mentioned by the participants. In the recent years the appearance of RTIs as a major problem of public health (due to increased sensitivity of the public and the officials), had led to increased number of individuals that have studied this field. Then these experts of RTIs became involved in various positions in decision making and executive jobs. This might have had effect in the reduction of RTIs in Iran. In this regard one of the participants stated: 

*“... In addition to these discussions, the use of specialists and educated manpower alongside decision-makers such as police,* *road safety commission and etc. has also been effective ...* *even some administrative posts during the years are assigned to educated and expert manpower in the field ...” (P2).*

## Discussion

As the result of the analysis of the interviews on the barriers to prevent RTIs, five main themes including structural barriers, organizational barriers and planning, socio-cultural barriers, scientific barriers, and inter-sector barriers and 22 sub-themes were identified. Furthermore, five general facilitators identified including: sensitization of the society and authorities, improving resources and infrastructure (software and hardware), increasing the attention to safety of vehicles and roads, increasing the information and awareness, and use of expert manpower.

The lack of traffic organization that is among the structural barriers, due to high repetition by the participants (almost all participants) and its importance was selected as the main barrier. Various organization are involved in RTIs and their prevention of which the most important ones are Ministry of Roads and Transportation, Ministry of Industries, Ministry of Health and Medical Education, Traffic Police, Ministry of Islamic Culture and Guidance, Ministry of Education, Legal Medicine Organization, Insurance Organization, Ministry of Justice, Ministry of Interior, the Red Crescent Organization, Emergency Services, Firefighting, Police, Broadcasting Organization, and the Judiciary branch [28]. Each of the mentioned organizations has specific responsibility, but as the participants acknowledged in the study, none of these organizations have sufficient authorities and resources to lead and manage RTIs consistently. In the countries succeeded in reducing the RTIs, usually an institution or organization with sufficient authority had the stewardship of the RTIs and other organizations are responsible for interaction and cooperation with it[47, 48]. For example, in Canada, the federal and provincial governments are leader at the levels of governmental territory in road safety. The federal government has commanding role in the transport system and with gathering information and conducting research is involved in the development and evaluation of this system. In the USA the general policies of transportation (railway, airway, sailing, and roads) are established by the Department of Transportation. But the safety rules of the roads particularly are released by the National Highway Traffic Safety Administration. In the Sweden the Swedish Transport Administration has all the responsibilities of policy making, implementing, monitoring the programs, road safety, and road maintenance. In the India the Ministry of Road Transport and Highways has the responsibility of establishing and executing the regulations, and monitoring them [47]. In the study conducted by Surrey *et al*. (2009), traffic police or the presidency were suggested as leading organization in the prevention of RTIs. Although in the recent years the traffic police have had a more active role in the management of RTIs and good progress has been made ​​in this area, the police have not adequate powers and resources to play the role of the leading organization. So it seems that, like most successful countries in the field of the prevention of RTIs, a committee of the representatives of the various organs of interest managed by the president should be established with a clear definition of the duties in order to play the leading role so that more appropriate results are achieved.

 Weak inter-sector cooperation and coordination were one of the main barriers that participants mentioned repeatedly. According to the WHO reports in 2004 and 2006, preventing RTIs is a collective responsibility and it would not be achieved except through the cooperation and coordination of different organizations and departments [49, 50]. This issue is also referred in the previous studies in Iran [25, 51, 52]. The main reason for this, as mentioned above, can be the existence of a lot of organizations are interested/involved in RTIs with unclear description of responsibilities and sometimes contradictory or parallel to each other. The absence of a leading organization also adds to the seriousness of this problem. Creating a lead organization might help to solve this problem to some extent.

Short-term and often inefficient planning was one of the most important barriers in the areas of organizational barriers mentioned by most participants. The plans on RTIs in Iran usually lack the strategic vision and face problems in implementation. One possible reason for this problem may be the reliance of the programs on the persons. Some body prepares a plan, mostly by his/her own knowledge and experience, and when he/she has no more the authority, the plans change. This vicious circle then repeats. Sweden has managed to reduce and control RTIs based on timed strategic planning and by following annual objectives so that  the symbol of this program is the program of Vision Zero [53, 54]. Many successful countries in this regard have had similar strategic plans for preventing the RTIs [55, 56]. In the report by the WHO in 2004, one of the proposed measures to reduce RTIs is strategic and appropriate planning [49]. Incorrect and short-term planning may be due to other barriers, which were noted in the study such as the lack of leading organization, unclear tasks and roles, policymakers’ attitudes and lack of information. In this regard we propose a long-term macro-level plan with mid-term and short-term objective in which the responsibilities of all involved parties are clearly defined. Then the plans would not person-reliant. 

The lack of basic and academic education was one of the major barriers in the area of scientific barriers mentioned by most participants. Studies have shown that public education alone does not reduce the losses or injuries of accidents [57, 58]. So the role of education and information dissemination and its value is diminished. The public education and awareness-raising which change the behaviors and the implementation of the relevant laws and regulations, might be effective together [59]. Education and public awareness-raising regarding the rules of the road and the increase of the acceptance of responsibility can be clearly effective. For example, people can be taught that what vehicle is safer for them to buy. Moreover, education can create the suitable atmosphere regarding the safety or acceptance of other interventions. In Iran, however, limited education is offered at schools on RTIs that its obvious example is the plan of the Police Collaborators [27], almost no education credit at the academic level is taught. Therefore, it is necessary that a series of public education (in the form of a single subject) for all academic disciplines will be considered. Like many countries of HICs, it is better to consider specialized fields at postgraduate level for the prevention of RTIs. As mentioned prior, one of the facilitators of RTI prevention is using expert manpower. The majority of current experts are graduates of foreign universities because the academic field is not available in Iran. Since studying in an abroad country is hard, it is better to establish the discipline in the domestic universities. 

Senior officials’ attitude, people’s attitudes, traditional view, people’s low awareness, and low demand for safety were five barriers in the field of socio-cultural area. It seems that one of the main factors resulting in these barriers is low awareness. These problems can be resolved with different interventions. One of the most effective interventions can mobilize the media and sharing some information regarding the prevention of RTIs. Nowadays, this strategy is used in different countries [60-63]. Designing and implementing this mobilization appropriately will lead to less-cost accessibility to a larger group of audience receiving educational messages. The success of such programs depends on the accompanying of the complementary school-based and society-based programs. The presence of health education specialists along with the media employees in the design process of these programs can increase the efficiency and effectiveness of these measures. According to the search results, currently no intervention in this regard is in action in Iran. Thus, to remove these barriers, at least designing a cohort study that measures the effect of the educational mobilization of the media on the prevention of RTIs can be effective.

The sensitization of society and the authorities on the intensity of the problem of RTIs was another facilitator of the prevention of RTIs mentioned by most participants. The reason for this, in the participants’ view, was the increased number of deaths and injuries resulting from RTIs, reports published at national and international level concerning RTIs in Iran, publishing, broadcasting reports of RTIs, especially during the New Year period on the severity of the problem of RTIs and other similar reasons. The sensitization of society and the authorities puts the RTIs on the agenda of the government and many interested organizations. Being the RTIs on the agenda can be effective in reducing them directly (planning programs and intervention to reduce them) and indirectly (commitment and its importance)[64, 65]. As noted in the study by the participants, the dissemination of reports and information can change authorities and people’s attitudes and sensitize them. As it worked when approximately 40 years ago the last report of the WHO on road safety was released, it created a significant change in the transportation safety experts’ view and understanding about the necessary measures to prevent accidents and their damages [49].

A limitation of this study is poor generalizability of the study results to other environments and countries. Yet, carrying out similar studies in other countries might be helpful in identifying the barriers and facilitators of the prevention of RTIs in other countries.

The results of the present study indicated that from the key experts and stakeholders’ viewpoint in the field of RTIs in Iran, the barriers of prevention of RTIs are more than their facilitators. Therefore, planning and paying more attention to remove these barriers and to promote the facilitators seems indispensable. Designating a leading organization with adequate authority and resources, designing a systematic model of strategic plan in the field of the prevention of RTIs, designing and developing effective and efficient educational programs aimed at changing people’s behavior, mobilizing the media in terms of information dissemination conceding the prevention of RTIs, and establishing a committee with the representatives of the various organs of interest with clear definition of the duties in the field of managing the RTIs might be helpful.

## References

[1] Azami-Aghdash S, Gorji HA, Sadeghi-Bazargani H, Shabaninejad H (2017). Epidemiology of road traffic injuries in Iran: based on the data from Disaster Management Information System (DMIS) of the Iranian Red Crescent. Iranian Red Crescent Medical Journal.

[2] Page L (2000). The new midwifery science and sensitivity in practice.

[3] Khorasani Zavareh D, Bohm K, Khankeh H, Talebian MT, Mohammadi R, Bigdeli M, Castren M (2015). Why should being visible on the road? A challenge to prevent road traffic injuries among pedestrians in Iran. J Inj Violence Res.

[4] Sadeghi-Bazargani H, Ayubi E, Azami-Aghdash S, Abedi L, Zemestani A, Amanati L (2016). Epidemiological patterns of road traffic crashes during the last two decades in Iran: a review of the literature from 1996 to 2014. Archives of trauma research.

[5] WHO (2015). Global status report on road safety 2015.

[6] Cunningham F (2005). Williams Obstetrics.

[7] Brewin M, Coggan C (2002). Evaluation of a New Zealand indigenous community injury prevention project. Inj Control Saf Promot.

[8] Bunn F, Collier T, Frost C, Ker K, Roberts I, Wentz R (2003). Area-wide traffic calming for preventing traffic related injuries. Cochrane Database Syst Rev.

[9] Dharmaratne SD, Ameratunga SN (2004). Road traffic injuries in Sri Lanka: a call to action. J Coll Physicians Surg Pak.

[10] Manno M, Rook A, Yano-Litwin A, Maranda L, Burr A, Hirsh M (2011). On the road with injury prevention--an analysis of the efficacy of a mobile injury prevention exhibit. J Trauma.

[11] Peden M (2005). Global collaboration on road traffic injury prevention. Int J Inj Contr Saf Promot.

[12] Pless B (2004). Road traffic injury prevention. BMJ.

[13] Naghavi M, Akbari M (2002). Injury epidemiology external caused of accident in Iran.

[14] Naghavi M, Jafari N, Alaeddini F, Akbari M (2004). Epidemiology of injuries due to external causes in the Islamic Republic of Iran.

[15] Rezaei S, Arab M, Karami Matin B, Akbari Sari A (2014). Extent, consequences and economic burden of road traffic crashes in Iran. J Inj Violence Res.

[16] Azami-Aghdash S, Najafzadeh MA, Heydari M, Rezapour R, Khasraghi JS, Derakhshani N (2018). Content-Analysis of text and video news of traffic accidents in Iran during the years 2001-2017. J Clin Res Gov.

[17] (2010). Statistical Yearbook of Road Maintenance and Transportation.

[18] Abedi L, Sadeghi-Bazargani H (2017). Epidemiological patterns and risk factors of motorcycle injuries in Iran and Eastern Mediterranean Region countries: a systematic review. Int J Inj Contr Saf Promot.

[19] Bakhtiyari M, Mehmandar MR, Mirbagheri B, Hariri GR, Delpisheh A, Soori H (2014). An epidemiological survey on road traffic crashes in Iran: application of the two logistic regression models. Int J Inj Contr Saf Promot.

[20] Azami-Aghdash S, Sadeghi-Bazarghani H, Rezapour R, Heydari M, Derakhshani N (2019). Comparative Study of Stewardship of Road Traffic Injuries Prevention with a Focus on the Role of Health System; Three Pioneer Countries and Three Similar to Iran. Bull Emerg Trauma.

[21] Ainy E, Soori H, Ganjali M, Baghfalaki T (2015). Eliciting road traffic injuries cost among Iranian drivers' public vehicles using willingness to pay method. Int J Crit Illn Inj Sci.

[22] Ayati I, Ghadriyan F, Ahadi M (2008). The Estimating cost of damage to vehicles in road accidents in Iran in 2004. J Transportation.

[23] Akbari ME, Naghavi M, Soori H (2006). Epidemiology of deaths from injuries in the Islamic Republic of Iran. East Mediterr Health J.

[24] Azami-Aghdash S, Sadeghi-Bazargani H, Shabaninejad H, Abolghasem Gorji H (2017). Injury epidemiology in Iran: a systematic review. J Inj Violence Res.

[25] Khorasani-Zavareh D, Mohammadi R, Khankeh HR, Laflamme L, Bikmoradi A, Haglund BJ (2009). The requirements and challenges in preventing of road traffic injury in Iran A qualitative study. BMC Public Health..

[26] Khorasani-Zavareh D, Shoar S, Saadat S (2013). Antilock braking system effectiveness in prevention of road traffic crashes in Iran. BMC Public Health..

[27] Soori H, Ainy E, Montazeri A, Omidvari S, Jahangiree AR, Shiran GR (2010). The role of pupil liaisons’ on traffic penalties and road traffic injuries. Payesh.

[28] Soori H, Ainy E, Movahedinejad A, Mahfozphoor S, Vafaee R, Hatamabadi H (2009). A practical model of political mapping in road traffic injury in Iran in 2008. Hakim Research Journal.

[29] Soori H, Nasermoadeli A, Movahedi M, Mehmandar M, Hatam Abady H, Rezazadeh Azari M (2009). The effect of mandatory seat belt use legislations on mortalities from road traffic injuries in Iran. Hakim Research Journal.

[30] Soori H, Royanian M, Zali A, Movahedinejad A (2009). Study of changes on road traffic injury rates, before and after of four interventions by Iran traffic police. Pejouhandeh.

[31] Ardalan A, Sepehrvand N, Pourmalek F, Masoumi G, Sarvar M, Mahmoudabadi A (2014). Deadly rural road traffic injury: a rising public health concern in IR Iran. Int J Prev Med.

[32] Azami-Aghdash S, Sadeghi-Bazarghani H, Heydari M, Rezapour R, Derakhshani N (2018). Effectiveness of Interventions for Prevention of Road Traffic Injuries in Iran and Some Methodological Issues: A Systematic Review. Bull Emerg Trauma.

[33] Thomson O'Brien MA, Oxman AD, Davis DA, Haynes RB, Freemantle N, Harvey EL (2000). Audit and feedback versus alternative strategies: effects on professional practice and health care outcomes. Cochrane Database Syst Rev.

[34] Gilson L, Erasmus E, Borghi J, Macha J, Kamuzora P, Mtei G (2012). Using stakeholder analysis to support moves towards universal coverage: lessons from the SHIELD project. Health Policy Plan.

[35] Gupta N, Fischer AR, van der Lans IA, Frewer LJ (2012). Factors influencing societal response of nanotechnology: an expert stakeholder analysis. J Nanopart Res.

[36] Tetali S, Lakshmi JK, Gupta S, Gururaj G, Wadhwaniya S, Hyder AA (2013). Qualitative study to explore stakeholder perceptions related to road safety in Hyderabad, India. Injury..

[37] Ghojazadeh M, Hajebrahimi S, Azami-Aghdash S, Pournaghi Azar F, Keshavarz M, Naghavi-Behzad M (2014). Medical students’ attitudes on and experiences with evidence-based medicine: a qualitative study. Journal of Evaluation in Clinical Practice.

[38] Ghojazadeh M, Azami-Aghdash S, Sohrab-Navi Z, Kolahdouzan K (2015). Cardiovascular patients' experiences of living with pacemaker: Qualitative study. ARYA Atheroscler.

[39] Graneheim UH, Lundman B (2004). Qualitative content analysis in nursing research: concepts, procedures and measures to achieve trustworthiness. Nurse Educ Today.

[40] Hsieh HF, Shannon SE (2005). Three approaches to qualitative content analysis. Qual Health Res.

[41] Byrne M (2001). Sampling for qualitative research. AORN journal.

[42] Cleary M, Horsfall J, Hayter M (2014). Data collection and sampling in qualitative research: does size matter?. J Adv Nurs.

[43] Higginbottom GMA (2004). Sampling issues in qualitative research. Nurse Researcher (through 2013).

[44] Grbich C (2007). Qualitative data analysis: An introduction.

[45] Hsieh HF, Shannon SE (2005). Three approaches to qualitative content analysis. Qual Health Res.

[46] Pope C, Ziebland S, Mays N (2000). Qualitative research in health care Analysing qualitative data. BMJ.

[47] Stevenson M, Thompson J (2014). On the road to prevention: road injury and health promotion. Health Promot J Austr.

[48] Al Turki YA (2014). How can Saudi Arabia use the Decade of Action for Road Safety to catalyse road traffic injury prevention policy and interventions?. Int J Inj Contr Saf Promot.

[49] WHO (2004). World report on road traffic injury prevention.

[50] Nisbeth O, Klausen K, Andersen LB (2000). Effectiveness of counselling over 1 year on changes in lifestyle and coronary heart disease risk factors. Patient Educ Couns.

[51] Haghparast-Bidgoli H, Hasselberg M, Khankeh H, Khorasani-Zavareh D, Johansson E (2010). Barriers and facilitators to provide effective pre-hospital trauma care for road traffic injury victims in Iran: a grounded theory approach. BMC Emerg Med..

[52] Khankeh HR, Mohammadi R, Ahmadi F (2007). Health care services at time of natural disasters: a qualitative study. Iran Journal of Nursing.

[53] Bergh T, Carlsson A, Larsson M (2003). Swedish vision zero experience. International journal of crashworthiness.

[54] Reichenbach M (Springer).

[55] Wegman F, Elsenaar P (1997). Sustainable solutions to improve road safety in the Netherlands.

[56] Elvik R (2001). Quantified road safety targets: an assessment of evaluation methodology.

[57] Duperrex O, Bunn F, Roberts I (2002). Safety education of pedestrians for injury prevention: a systematic review of randomised controlled trials. BMJ.

[58] Ker K, Roberts I, Collier T, Renton F, Bunn F (2003). Post-licence driver education for the prevention of road traffic crashes. Cochrane Database Syst Rev.

[59] Elvik R (2004). Handbook of road safety measures Amsterdam.

[60] Stead M, Tagg S, MacKintosh AM, Eadie D (2005). Development and evaluation of a mass media Theory of Planned Behaviour intervention to reduce speeding. Health Educ Res.

[61] Yadav RP, Kobayashi M (2015). A systematic review: effectiveness of mass media campaigns for reducing alcohol-impaired driving and alcohol-related crashes. BMC Public Health..

[62] Hutchinson TP, Wundersitz LN (2011). Road safety mass media campaigns: why are results inconclusive, and what can be done?. Int J Inj Contr Saf Promot.

[63] Elder RW, Shults RA, Sleet DA, Nichols JL, Thompson RS, Rajab W (2004). Task Force on Community Preventive Services Effectiveness of mass media campaigns for reducing drinking and driving and alcohol-involved crashes: a systematic review. Am J Prev Med.

[64] Albalawi Y, Sixsmith J (2015). Agenda Setting for Health Promotion: Exploring an Adapted Model for the Social Media Era. JMIR Public Health Surveill.

[65] Setyarahajoe R (2012). Order establishing traffic rules with agenda setting model in surabaya. Academic Research International.

